# Sea cucumber-derived compounds for treatment of dyslipidemia: A review

**DOI:** 10.3389/fphar.2022.1000315

**Published:** 2022-09-14

**Authors:** Ping Lin, Nuo Shen, Fan Yin, Shou-Dong Guo

**Affiliations:** Institute of Lipid Metabolism and Atherosclerosis, Innovative Drug Research Centre, School of Pharmacy, Weifang Medical University, Weifang, China

**Keywords:** sea cucumber, bioactive component, lipid-lowering, mechanisms of action, NALFD, cardiovascular disease

## Abstract

Dyslipidemias are disorders of plasma levels of lipids, such as elevated levels of total cholesterol and triglyceride, that are associated with various human diseases including cardiovascular disease (CVD) and non-alcoholic fatty liver disease (NAFLD). Statins are the first-line drugs for treatment of dyslipidemia. However, a substantial proportion of patients cannot reach the recommended LDL-c level even with the highest tolerated doses of statins, and there is no available drug specifically for NAFLD therapy. Sea cucumbers are one of the widely distributed invertebrates, and are an important resource of food and medicine. Sea cucumbers have many valuable nutrients including saponins, fatty acids, phospholipids, cerebrosides, sulfated polysaccharides, as well as proteins and peptides. In recent years, these natural products derived from sea cucumbers have attracted attentions for treatment of CVD and NAFLD because of their lipid-lowering effect and low toxicity. However, the hypolipidemic mechanisms of action and the structure-activity relationship of these bioactive components have not been well-documented in literature. This review article summarizes the signaling pathways and the potential structure-activity relationship of sea cucumber-derived bioactive compounds including saponins, lipids, carbohydrates as well as peptides and proteins. This article will provide information useful for the development of sea cucumber-derived lipid-lowering compounds as well as for investigation of hypolipidemic compounds that are derived from other natural resources.

## Introduction

Lipid homeostasis is finely tuned by multiple systems and organs, which interact with each other through cross-talks via cellular signaling upon molecular stimulation. Peroxisome proliferator-activated receptors (PPARs) are lipid sensors and play key roles in lipid homeostasis. PPARα is primarily expressed in liver, brown adipose tissue, heart, and muscle tissue. It is the master regulator of lipid metabolism via modulating fatty acid (FA) transport and β-oxidation. PPARγ is expressed mainly in adipose tissue, where it regulates adipogenesis (Tian et al., 2020). The wingless-type MMTV integration site (WNT)/β-Catenin pathway plays a key role in regulating adipogenesis. Glycogen synthase kinase-3β can phosphorylate β-Catenin, thereby causing degradation of β-Catenin. Furthermore, the frizzled receptor (Fz) and lipoprotein receptor-related protein (LRP)5/6 coreceptors located at the cell membrane can bind to WNT10b, destructing the activated degradation of β-Catenin, thereby promoting the accumulation of β-Catenin in cytoplasm and the subsequent nuclear translocation. In the nucleus, β-Catenin binds to T cell factor lymphoid enhancer factor family to activate downstream genes, such as cyclin D1 and C-myc, leading to inhibition of the expression of PPARγ and CCAAT/enhancer binding protein-α (C/EBPα) (Christodoulides et al., 2009; Lee et al., 2010; Xu et al., 2014; Xu et al., 2015a). Additionally, accumulating evidence have demonstrated that gut microbiota consisted of trillions of bacteria affect host lipid homeostasis (Aron-Wisnewsky et al., 2021; de Vos et al., 2022).

Dyslipidemias are disorders of plasma levels of lipids, such as elevated levels of total cholesterol (TC) and triglyceride (TG), that are associated with various human diseases including cardiovascular disease (CVD) and fatty liver diseases (Pirillo et al., 2021). Accumulating evidence have demonstrated that hypercholesteremia, especially elevated level of low-density lipoprotein (LDL) cholesterol (LDL-c), is the major risk factor for CVD (Atar et al., 2021). Furthermore, hypertriglyceridemia is a key risk factor of the residual CVD and non-alcoholic fatty liver disease (NAFLD) (Heeren and Scheja, 2021; Zhang B. H. et al., 2022). According to the World Health Organization report in 2021, CVD remains the leading cause of human death and accounts for approximately 32% of the total deaths in 2019 (World Health Organization, 2021). NAFLD is characterized by the accumulation of TG and cholesterol in the liver and has a global prevalence of 25% (Powell et al., 2021). Lipid-lowering therapy is an effective strategy for prevention and/or treatment of CVD as well as NAFLD that are induced by dyslipidemia (Beshir et al., 2021; Ferraro et al., 2022). Statins are the first-line drugs for treatment of dyslipidemia. However, a substantial proportion of patients cannot reach the recommended LDL-c level even with the maximum tolerated doses of statins (De Backer et al., 2019). Furthermore, the overall efficacy of non-statin drugs on CVD outcomes is much less robust than that of statins (Visseren et al., 2021), and there is no available drug specifically for NAFLD therapy. In recent years, natural products have attracted attentions for treatment of CVD and NAFLD due to their powerful hypolipidemic effects and low toxicity (Singh and Sashidhara, 2017; Li et al., 2022).

Sea cucumbers are one of the widely distributed invertebrates, and are an important resource for food and medicine. The idea of “medicine and food are homologous (药食同源)” has been widely accepted in Asia, especially in China, and this idea is spreading all over the world. As reviewed previously, sea cucumbers have many valuable nutrients including vitamins, minerals, triterpene glycosides (saponins), sulfated polysaccharides, sterols, phenolics, cerebrosides, peptides, FA, and others. These components show various bioactivities such as anti-angiogenic, anti-tumor, anticoagulant, anti-hypertension, anti-inflammatory, anti-oxidant, antithrombotic, antimicrobial, immunomodulatory, and wound healing functions (Bordbar et al., 2011; Khotimchenko, 2018). Diets containing sea cucumber (*Isostichopus badionotus*) meals can reduce serum levels of TC and TG in young rats via modulating the expression of multiple genes including sterol regulatory element-binding transcription factor (SREBP), 3-hydroxy-3-methyl-glutaryl-CoA reductase (HMGCR), and liver X receptor (LXR) that are associated with lipogenesis (Olivera-Castillo et al., 2013). In the past decades, the lipid-modulatory mechanisms of action of the compounds derived from sea cucumbers have been understood. Additionally, novel technologies, such as liquid chromatography-tandem mass spectrometry (LC-MS/MS), are applied for the detection of metabolites of sea cucumbers (Wang et al., 2020; Zhao et al., 2020; Savarino et al., 2021). These advances make it possible to discuss the structure-activity relationship of these compounds obtained from sea cucumber. In this article, we summarize the lipid-lowering mechanisms of action and the potential structure-activity relationship of sea cucumber-derived bioactive components including saponin, lipid, long chain base, carbohydrate, peptide and protein. The related literature used in this article were mainly obtained as search results from PubMed using “sea cucumber and lipid” as keywords.

## The lipid-modulatory mechanisms of sea cucumber-derived compounds

### Saponin

**FIGURE 1:**
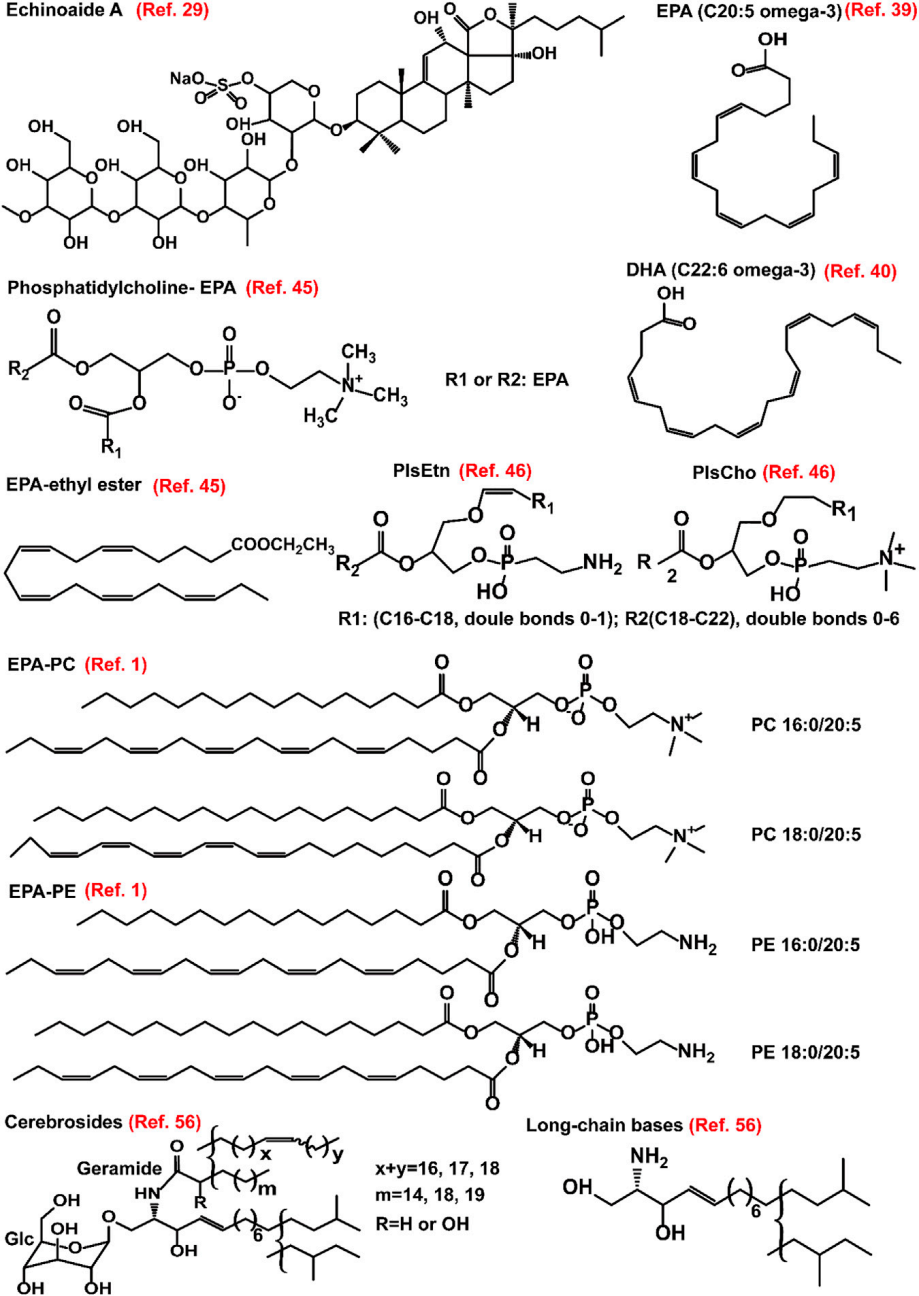
Structure of representative small molecules that are derived from sea cucumbers. These small molecules are found to modulate hyperlipidemia in different models including non-alcoholic fatty acid disease and atherosclerosis. EPA, eicosapentaenoic acid; DHA, docosahexaenoic acid; PC, phosphatidylcholine; PE, phosphatidylethanoamine; PlsCho, plasmanyl phosphatidylcholine; PlsEtn, plasmenyl phosphatidylethanoamine.

Saponin is one of the most important secondary metabolites and bioactive constitutes of sea cucumbers (Meng et al., 2018). In a comparative study, saponins derived from *Cucumaria frondosa* show better lipid-lowering activity compared to other components of sea cucumber including polysaccharides, collagen peptides, dregs, or non-saponin residues in rats. The underlying mechanism of action is associated with inhibition of the activity of pancreatic lipase, which is responsible for hydrolysis of dietary fat in the small intestine (Hu X. et al., 2012; Hu X. Q. et al., 2012). The crude saponins of sea cucumbers are generally extracted with 60% ethanol, and the obtained mixture can be further extracted with water-saturated *n*-butanol to obtain the final crude extracts that are consisted approximately 66% of saponins. These extracts obtained from sea cucumbers, such as *Thelenota ananas*, *Pearsonothuria graeffei* Semper (Holothuriidae), and *Holothuia fuscogliva*, significantly downregulate the activity of pancreatic lipase *in vitro*. Of note, the major bioactive component of saponin, echinoside A (Figure 1) accounts for 35.6% of the above water-saturated *n*-butanol dissolved components and has a 50% inhibitory concentration of 0.76 μM for pancreatic lipase *in vitro* (Guo et al., 2016). The 60% ethanol extract of *P. graeffei* reduces the body weight, serum levels of TC, TG, and LDL-c, and hepatic TC and TG in C57BL/6 mice fed with a high-fat diet. Mechanistically, this extract upregulates the LXR-β signaling molecules including LXR-β, ATP-binding cassette transporter (ABC) G1, and cholesterol 7-α hydroxylase (CYP7A1). Importantly, the bioactive component echinoside A acts not only by upregulating the expression of LXR-β but also via enhancing the expression of LXR-α, ABCG1, apolipoprotein (apo) E, and CYP7A1 in HepG2 cells at the dose of 2.5 μM (Guo et al., 2016). These data suggest that saponins, such as echinoside A, may alleviate hyperlipidemia via promoting the conversion of cholesterol to bile acid as well as lipid excretion.

**FIGURE 2:**
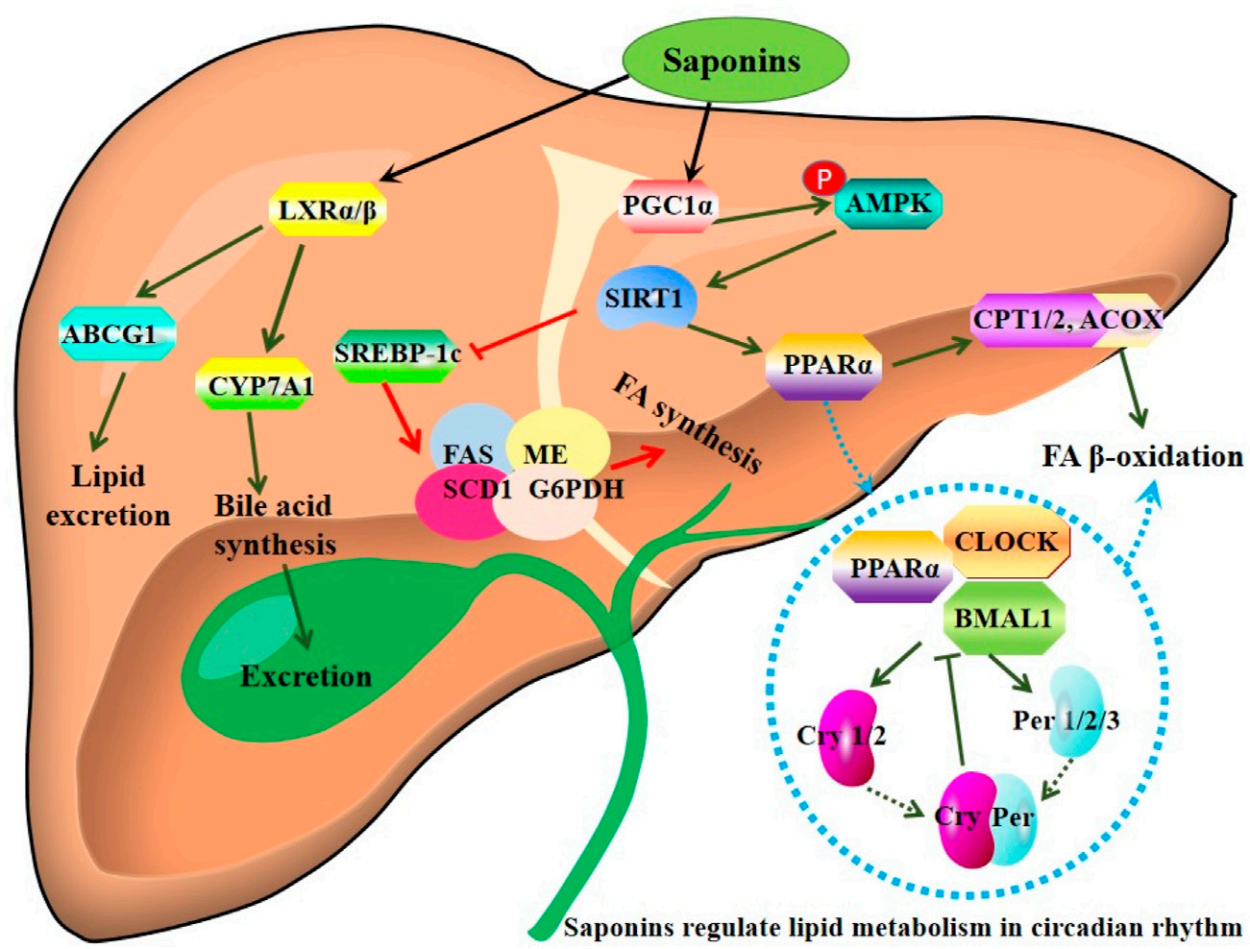
Mechanisms of action of saponins derived from sea cucumbers. Saponins stimulate LXR and AMPK/PPARα signaling pathways, thereby promoting lipid expression and fatty acid (FA) β-oxidation. Of note, these saponins regulate lipid metabolism-related genes in circadian rhythm. ABCG1: ATP-binding cassette transporter G1; ACOX1: acyl-CoA oxidase 1; AMPK: AMP-activated protein kinase; Cry: cryptochrome gene; CYP7A1: cholesterol 7-α-hydroxylase A1; FAS: fatty acid synthase; G6PDH: glucose-6-phosphate dehydrogenase; LXR: liver X receptor; ME: malic enzyme; CPT1: carnitine palmitoyl transferase 1; PGC-1α: peroxisome proliferator-activated receptor-γ co-activator 1α; Per: period gene; PPARα: peroxisome proliferator activated receptor α; SCD-1: Stearoyl-CoA desaturase-1; SIRT1: Sirtuin 1; SREBP-1c: sterol regulatory element-binding protein-1c. All the abbreviations are applicable for the rest Figures.

Furthermore, sea cucumber saponins reduce lipogenesis and promote FA β-oxidation via inhibiting SREBP-1c and enhancing the expression of PPARα and acyl-CoA oxidase 1 (ACOX1), respectively, thereby improving lipid deposition in Sprague-Dawley (SD) rats and C57BL/6 mice (Hu et al., 2010; Guo et al., 2018; Meng et al., 2018). Saponins inhibit the activity and mRNA expression of lipogenic enzymes including fatty acid synthase (*FAS*), malic enzyme, and glucose-6-phosphate dehydrogenase (*G6PDH*) in the liver of mice fed with the diet containing 1% OA and 0.05% saponins (Hu et al., 2010). In combination with eicosapentaenoic acid (EPA)-enriched phospholipids, sea cucumber saponins further reduce hepatic TG partially by enhancing the expression of PPARα. Furthermore, this combination shows better effect on improving glucose intolerance and systematic insulin sensitivity than monotherapy (Han et al., 2019). Interestingly, sea cucumber saponin treatment induces changes of lipid metabolism-related genes such as *PPARα*, *SREBP-1c*, carnitine palmitoyl transferase (*CPT*), and *FAS* in rhythm, suggesting saponin may modulate lipid metabolism by regulating the clock genes such as *CLOCK* and *BMAL1* in the ICR male mice fed with 0.03% sea cucumber saponin in regular chow (Wen et al., 2014). The major bioactive component of saponin, echinoside A, also regulates the expression of some key genes involved in lipid metabolism in a diurnal manner (Wen et al., 2016). The mechanisms of action of saponins are summarized in Figure 2.

### FA and phospholipid

Exogenous molecules can modulate lipid homeostasis by different signaling pathways. Exogenous lipids are recycled and/or degraded and participate in the formation of lipid raft, thereby affecting raft-related signaling pathways (Abumrad et al., 2012; Duan et al., 2012). Sea cucumber is a valuable food of FAs as well as other nutritional phospholipids (Roggatz et al., 2016; Roggatz et al., 2018). In the sea cucumber *Athyonidium chilensis*, saturated FAs are predominant in the tubule phospholipids (40.7%), while monounsaturated FAs account for approximately 42.0% and 38.0% of the phospholipids in the internal organs and body wall, respectively. The major polyunsaturated FAs are C20: 2ω-6 FA, arachidonic acid (C20: 4ω-6), and EPA (C20-5ω-3) (Figure 1) (Careaga et al., 2013). Furthermore, the presence of the odd carbon saturated FAs may be originated from the detritus, which is a part of the diet of sea cucumber (Careaga et al., 2013). Different solvents have distinct ability for extraction of FAs, and water is found to have a higher efficiency for extraction of docosahexaenoic acid (C22:6, Figure 1) compared to phosphate buffer saline, methanol, or ethanol (Fredalina et al., 1999). Accumulating evidence have demonstrated the health-beneficial effects of polyunsaturated FAs for treatment of lipid disorders (Djuricic and Calder, 2021; Mitrovic et al., 2022). Therefore, FAs, especially the n-3 polyunsaturated FAs that are enriched in sea cucumbers have potential application in pharmaceutical industries for intervention of dyslipidemias.

**FIGURE 3:**
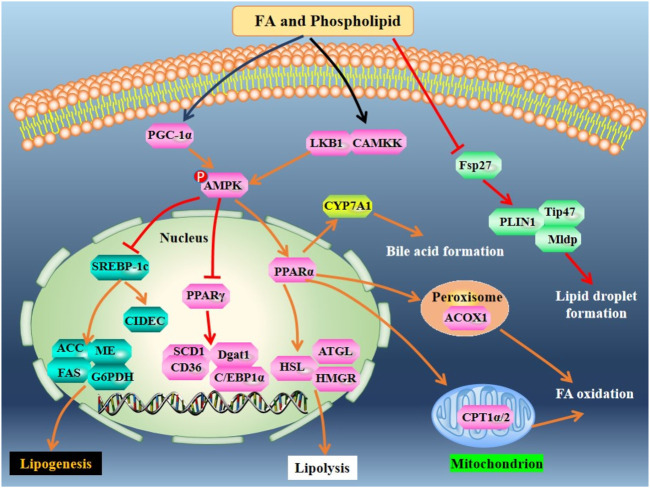
Mechanisms of action of FA and phospholipid derived from sea cucumbers. CAMKK, Ca^2+^-dependent kinase; C/EBPs: CCAAT/enhancer binding proteins; CIDEC: cell-death-inducing DFFA-like effector c; Dgat1, diacylglycerol acyltransferase 1; Fsp27, fat-specific protein 27; HMGR: hydroxymethyl glutaric acid acyl; LKB1, liver kinase B1; Mldp, perilipin5; PLIN1, perilipin1; Tip47, perilipin 3. All the abbreviations are applicable for the rest Figures.

Some EPA-enriched phospholipids are shown in Figure 1. EPA-enriched phospholipids reduce hepatic TG and TC in orotic acid-induced SD rats with NAFLD via enhancing PPARα-mediated FA β-oxidation. Furthermore, these 1% EPA-enriched phospholipids promote the expression of ACOX1 but not CPT-1 and CPT-2 (Guo et al., 2018). In rats, EPA-enriched phosphatidylcholine (EPA-PC) (80 mg/kg) attenuates NAFLD induced by 1% orotic acid via suppressing the mRNA expression of *HMGCR* and increasing the expression of sterol carrying protein 2, thereby inhibiting cholesterol synthesis and improving fecal cholesterol excretion, respectively. The underlying mechanisms are associated with the upregulation of PPARα and adenosine monophosphate activated protein kinase (AMPK) as well as its upstream modulators including liver kinase B1 and Ca^2+^-dependent kinase (Liu et al., 2017). A recent study demonstrated that PC contained in EPA-PC and PE contained in EPA-enriched phosphatidylethanoamine (EPA-PE) directly bind to and activate PPARα and PPARγ. In mouse hepatocytes and liver, 0.3% EPA-PC and 0.3% EPA-PE reduce lipid accumulation via enhancing PPARα-mediated FA β-oxidation. Although EPA-PC and EPA-PE (200 μg/ml) promote the conversion of preadipocyte to mature adipocyte in a 3T3-L1 cell model, they reduce phosphorylation of PPARγ at Ser273 *in vivo*, which may partially explain the reductions in the weight of adipose and the size of adipocyte (Tian et al., 2020). Furthermore, EPA-enriched phospholipids isolated from sea cucumber *C. frondosa* suppress lipid accumulation in mouse liver and white adipose via inhibiting the expression of lipiddroplet associated protein FSP27 and enhancing the expression of lipolysis genes including hormone-sensitive lipase (*HSL*), adipose triglyceride lipase (*ATGL*) as well as the lipogenesis gene *PPARγ* in the white adipose of male C57BL/6J mice fed with high-sucrose diet (Zhang et al., 2020). However, EPA-PC has no effect on FA profiles in the brain (Wen et al., 2018). Sea cucumbers are rich in ether-linked phospholipids including plasmenyl phosphatidylethanoamine (PlsEtn) and plasmanyl phosphatidylcholine (PlsCho) (Figure 1). PlsEtn accounts for >83% of the total PE, and PlsCho accounts for >59% of the total PC in sea cucumber. Both 0.3% PlsEtn and 0.3% PlsCho, especially PlsEtn, significantly reduce hepatic TC and TG in C57BL/6N mice with alcoholic liver disease induced by ethanol gavage. Mechanistically, PlsEtn and PlsCho inhibit FA uptake and TG synthesis via downregulation of the mRNA expression of hepatic cluster of differentiation 36 (*CD36*) and diacylglycerol acyltransferase 1, respectively. Additionally, PlsCho enhances FA oxidation via increasing the mRNA expression of *CPT-1α* in the liver of mice (Wang et al., 2022). The mechanisms of action of FAs and phospholipids derived from sea cucumbers are summarized in Figure 3.

### Cerebroside

The structure of some glucocerebroside molecular species has been characterized by different groups (Figure 1) (Yamada et al., 2002; Kisa et al., 2005; Yamada et al., 2005). Except for the classical column separation in combination with high-performance liquid chromatography, cerebrosides can be isolated using high speed counter-current chromatography (Xu et al., 2013). Sea cucumber cerebrosides isolated from *C. frondosa* contain approximately 48.0% of EPA. These lipids in liposome forms have particle sizes ranging from 169 to 189 nm and can efficiently penetrate the cell membrane in an M cell monolayer model as well as in a Caco-2 cell monolayer model (Du et al., 2016). The cerebrosides isolated from *Stichopus japonicus* can be absorbed *in vivo*, and then they are incorporated into ceramides, thereby improving the skin barrier function and increasing the cecal content of short-chain FA (SCFA) (Duan et al., 2016). A recent study indicates that liposomes derived from sea cucumbers is safe even at the concentration of 0.1 mg/ml (Mecheta et al., 2020). These data suggest that sea cucumber-derived cerebrosides can be explored as drug-loading nanoparticles.

**FIGURE 4:**
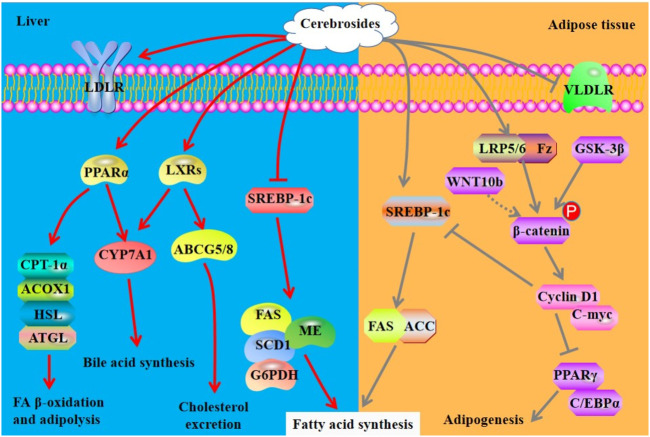
Mechanisms of action of cerebrosides derived from sea cucumbers. ACC, acetyl-CoA carboxylase; ATGL: adipose triglyceride lipase; GSK-3β, glycogen synthase kinase-3β; HSL, hormone sensitive lipase; LRP, lipoprotein receptor related protein; VLDLR, very low-density lipoprotein receptor; WNT10b, wingless-type MMTV integration site10b. All the abbreviations are applicable for the rest Figures.

In an orotic acid-induced NALFD model, cerebrosides obtained from *Acaudina molpadioides* increase serum TG, but reduce liver index and hepatic TG. Mechanistically, these cerebrosides reduce the activities and expression of lipogenic enzymes including FAS, malic enzyme, and G6PDH, that are the target genes of SREBP-1c. Furthermore, the mRNA expression of *SREBP-1c* and the activity of microsomal triglyceride transfer protein are inhibited by cerebrosides at 0.006% in the liver of rats (Zhang et al., 2012). As a diet supplement, the cerebroside isolated from sea cucumber *A. molpadioides*, designated as AMC-2, can reduce hepatic TC and TG via down-regulating the activity and mRNA expression of stearoyl-CoA desaturase (*SCD*) in rats with NAFLD (Xu et al., 2011). In apolipoprotein E-deficient mouse, an atherosclerosis model, cerebrosides isolated from sea cucumber *A. molpadioides* reduce the formation of atherosclerotic plaques, serum levels of TC and LDL-c, and hepatic TC and TG when they are added to food at the dose of 0.06%. Mechanistically, cerebroside treatment promotes the expression of LDL receptor, CYP7A1 and ABCG5/G8, thereby promoting reverse cholesterol transport. Furthermore, these compounds improve FA oxidation via enhancing the expression of PPARα, CPT-1α, and ACOX1, and suppress lipogenesis by inhibiting the expression of SREBP-1c and its target genes including *FAS* and *SCD1* (Zhang et al., 2018). The cerebrosides obtained from sea cucumber *A. molpadioides* also exhibit powerful effects on reduction of fat weight and serum and hepatic levels of TG via inhibiting the enzymatic activity of FAS and malic enzyme, the content of CPT, and the mRNA expression of *SREBP-1c* and *FAS*, in the liver of C57BL/6J mice fed with a diet containing 0.025% of cerebrosies. Furthermore, these cerebrosides significantly decrease the mRNA expression of very low-density lipoprotein receptor and lipoprotein lipase (*LPL*) and increase the expression of *SREBP-1c*, *FAS*, *ATGL*, and acetyl CoA carboxylase (*ACC*) in the white adipose tissue (Liu et al., 2015). In 3T3-L1 cells, 250 μg/ml cerebrosides isolated from sea cucumber *C. frondosa* promote the nuclear translocation of β-Catenin and the expression of its target genes such as cyclin D1 and C-myc, and the expression of Fz and LRPs, thereby suppressing the expression of PPARγ and C/EBPα (Xu et al., 2015a). Cerebrosides have different effects on modulation of adipocyte differentiation both *in vitro* and *in vivo* via regulation of related signaling pathways in a different manner. The mechanisms of action of cerebrosides derived from sea cucumber are summarized in Figure 4.

### Long-chain base

**FIGURE 5:**
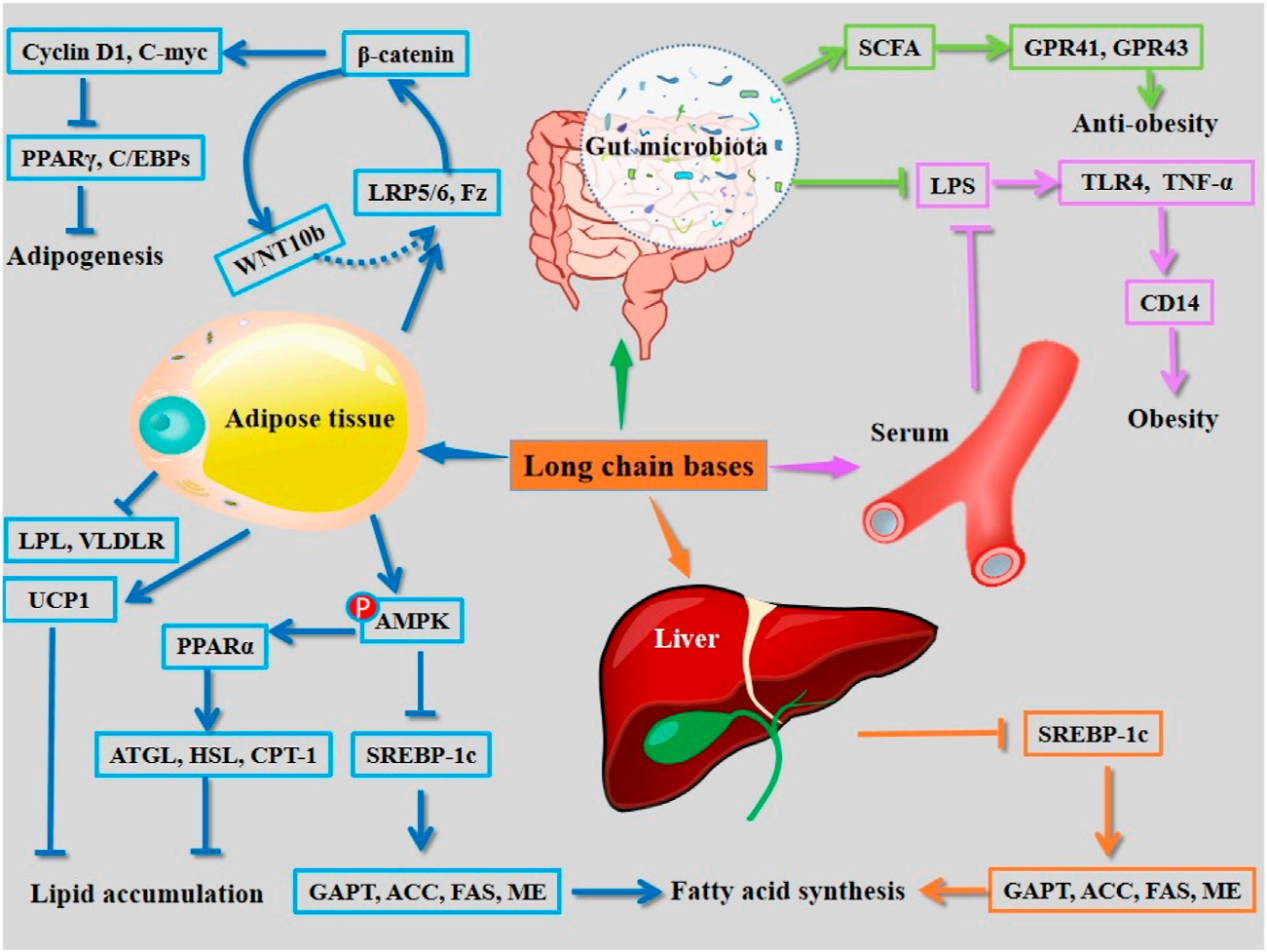
Mechanisms of action of long chain bases derived from sea cucumbers. CD14, cluster of differentiation 14; GPAT, glycerol-3-phosphate acyl-transferase; GPR, G protein-coupled receptor; LPL, lipoprotein lipase; LPS, lipopolysaccharide; SCFA, short chain fatty acid; TLR4, Toll-like receptor 4; TNF-α, tumor necrosis factor α; UCP1, uncoupling protein 1. All the abbreviations are applicable for the rest Figures.

Long-chain bases can be obtained by acid hydrolysis of cerebrosides (using 10% HCl) (Figure 1), which are the main active structural units of cerebrosides for intervention of hyperlipidemia (Liu et al., 2015). Long-chain bases are found to ameliorate obesity by multiple pathways. The long-chain bases isolated from *A. molpadioides* significantly reduce the body weight, fat weight, plasma levels of TG, TC, LDL-c, glucose, leptin, and insulin, and increase the levels of plasma high density lipoprotien cholesterol (HDL-c), fecal SCFAs including acetate, propionate, and butyrate, as well as the expression of SCFAs-mediated G-protein-coupled receptors in mice. In the gut, long-chain bases induce reductions in *Firmicultes* and *Actinobacteria* phylum, and obesity-associated bacteria including *Desulfovibro*, *Bifidobacterium*, and *Romboutsia* at the genus level. They increase the abundance of *Bacteroidetes*, *Proteobacteria*, and *Verrucomicrobia* phylum, and the SCFAs-producing bacteria including *Bacteroides*, *Lactobacillus*, and *Lachnospiraceae_NK4A136_group* at the genus level (Hu et al., 2019). Phosphorylation of AMPK stimulates the phosphorylation of ACC, causing down-regulation of the activity of ACC and the expression of lipogenesis related enzymes including FAS. Furthermore, activation of AMPK through phosphorylation un-regulates lipidolysis via enhancing the expression of HSL and CPT-1 (Tian et al., 2016; Aslam and Ladilov, 2022). Of note, 50 μg/ml and 100 μg/ml of long-chain bases obtained from *C. frondosa* inhibit adipogenesis in 3T3-L1 pre-adipocytes via enhancing the phosphorylation of AMPK and ACC. Furthermore, they inhibit the transcriptional factors, such as C/EBPs and PPARγ, and activate WNT/β-catenin and its target genes including cyclin D1 and C-myc, thereby inhibiting adipocyte differentiation (Tian et al., 2016). The mechanisms of action of long chain bases derived from sea cucumber are summarized in Figure 5.

### Carbohydrate

**FIGURE 6:**
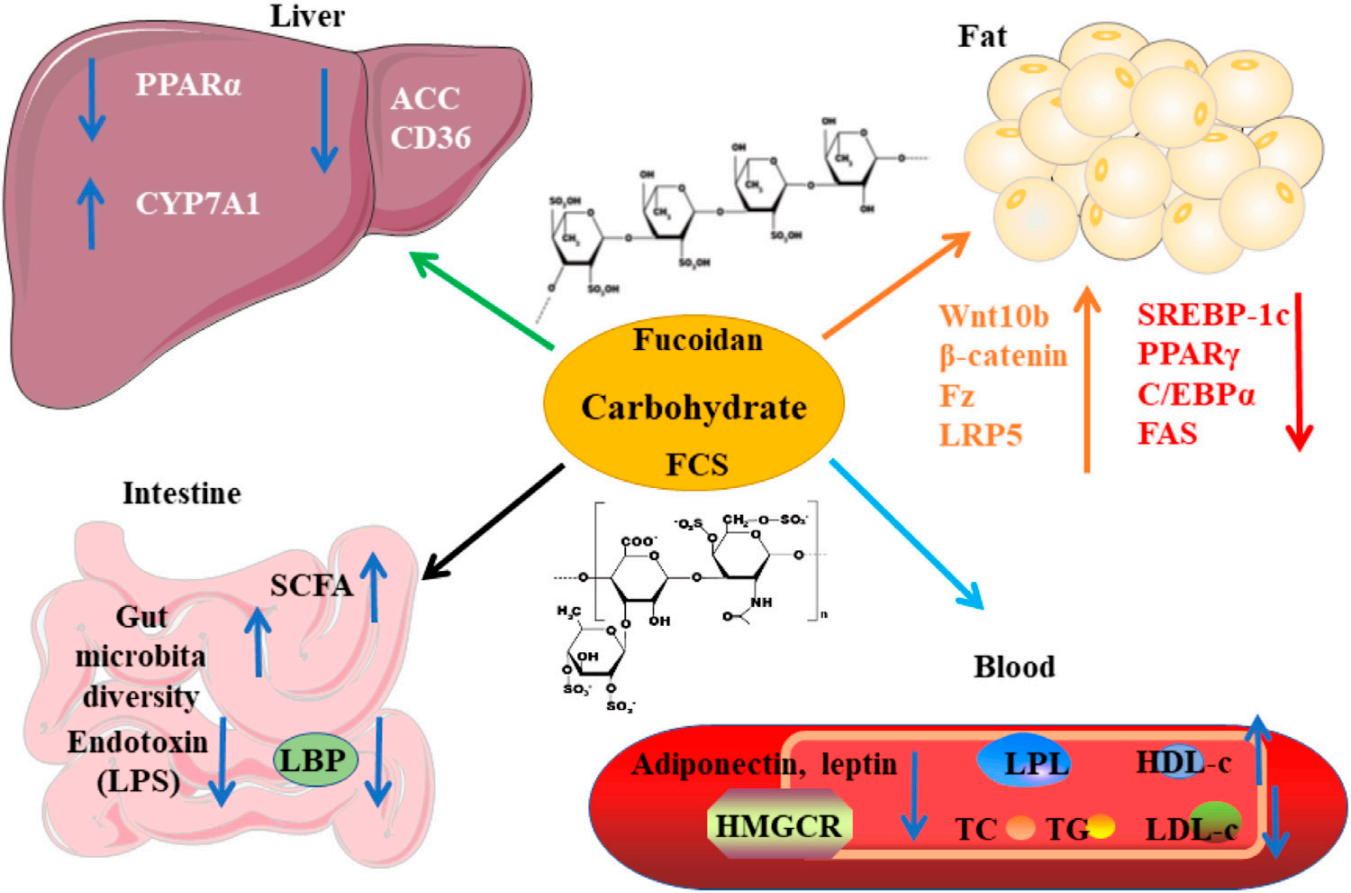
The mechanisms of action of carbohydrates derived from sea cucumbers. These polysaccharides include fucoidan and fucosylated chondroitin sulfate (FCS). HDL-c, high-density lipoprotein cholesterol; LBP, lipopolysaccharide binding protein; LDL-c, low-density lipoprotein cholesterol; LPS, lipopolysaccharide; TC, total cholesterol; TG, triglyceride. All the abbreviations are applicable for the rest Figures.

**FIGURE 7:**
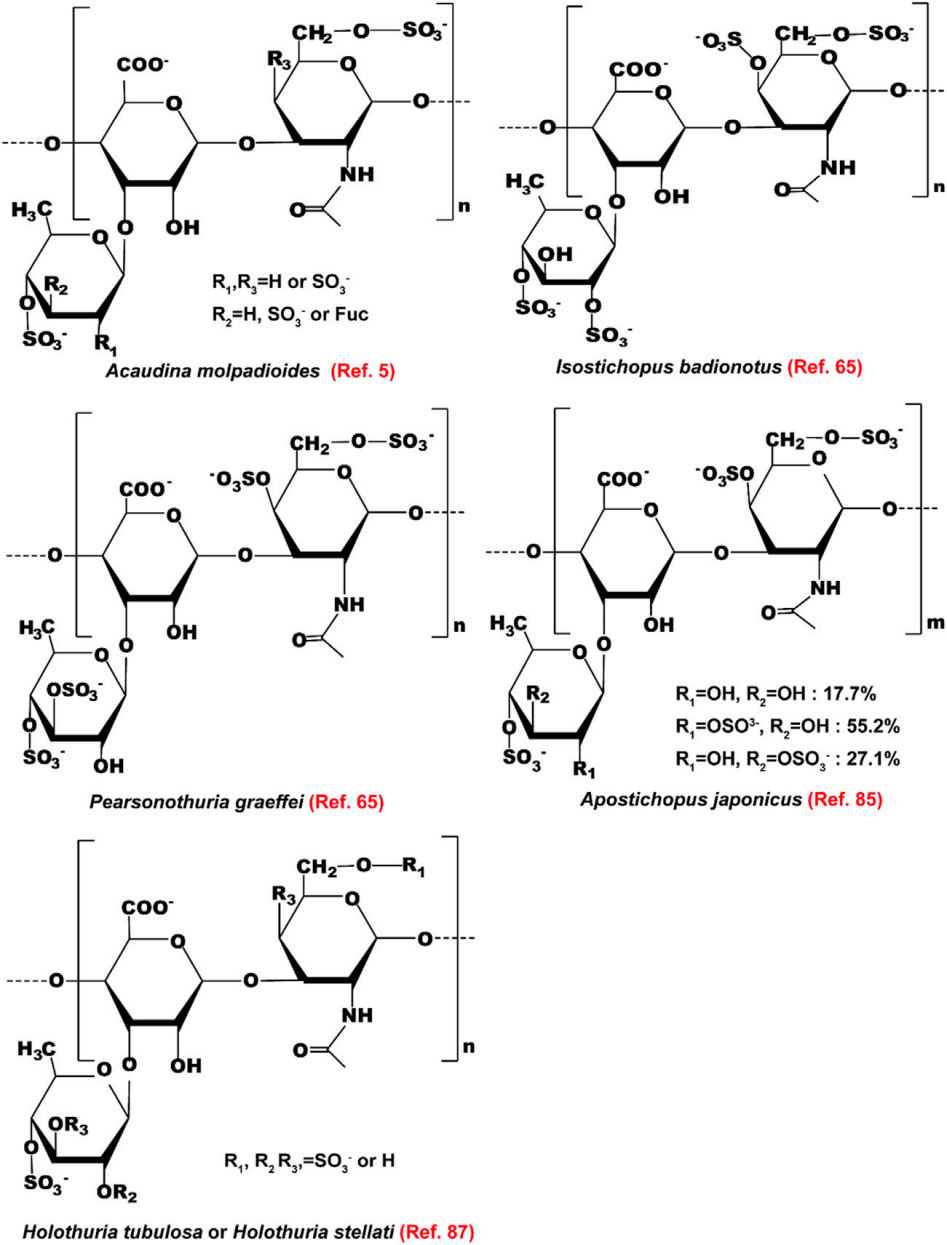
The structure of fucosylated chondroitin sulfates extracted from different sea cucumbers.

Polysaccharides of sea cucumber reduce serum levels of TG, TC, and LDL-c, and increase the level of HDL-c in rats (Hu X.Q. et al., 2012; Liu et al., 2012). The health-beneficial activities including the hypolipidemic effect of fucosylated chondroitin sulfate (FCS) isolated from sea cucumbers have been reviewed recently by Xu et al. (2022). Here, we summarize the mechanisms of action and the structure-activity relationship of FCS as lipid-lowering agents (Figure 6). The structure of these FCSs extracted from different sea cucumbers are shown in Figure 7. FCS isolated from *A. molpadioides* elevates the expression of Wnt/β-Catenin signaling molecules, such as Wnt10b, β-Catenin, Fz, and LRP5, leading to down-regulation of the transcriptional factors including SREBP-1c, PPARγ, and C/EBPα, thereby exhibiting anti-adipogenic effects in 3T3-L1 cells and mice (Xu et al., 2015b). Of note, the FCS isolated from *P. graeffei* with 3,4-O-disulfated fucose branches exhibits a more powerful effect in lipid-lowering than the FCS obtained from *I. badionotus* with 2,4-O-disulfated fucose branches (Chen et al., 2012; Wu et al., 2016). These data suggest that the substitution position of the sulfate group at the branched fucosyls plays a key role in the hypolipidemic effect of FCS. Furthermore, the FCS (40 mg/kg) with a molecular weight (Mw) of 4.3 kDa obtained via degradation of FCS of *I. badionotus* shows hypolipidemic effect in C57BL/6 mice fed with a high-fat diet by down-regulating the mRNA expression of *FAS* and *PPARγ* in adipose tissues (Li et al., 2018a). These data suggest that depolymerized FCS with low Mw maintains the lipid-lowering effect of its native FCS. However, the influence of Mw on the hypolipidemic effect of FCS need to be further investigated by comparative studies.

**FIGURE 8:**
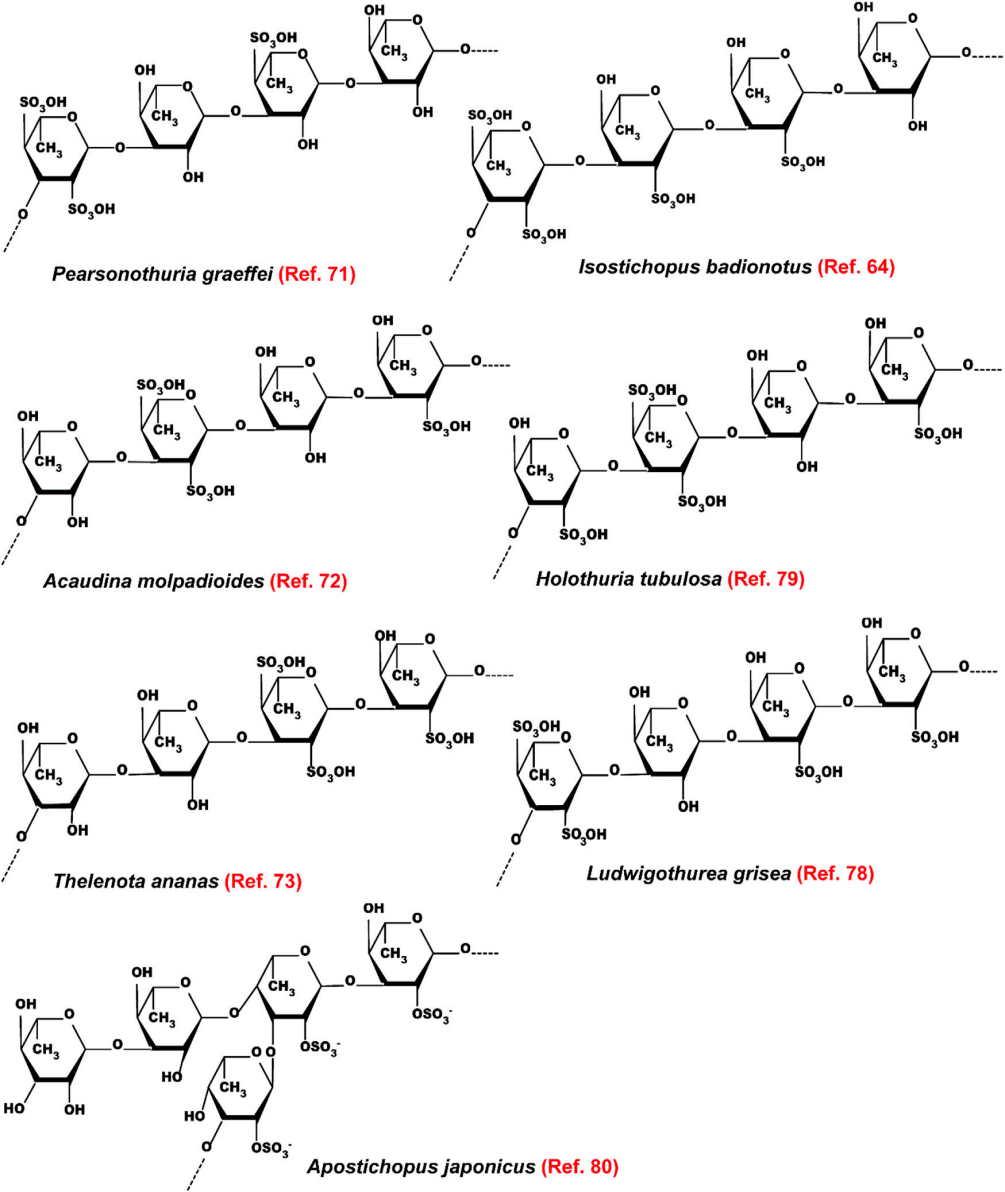
The structure of fucoidans extracted from different sea cucumbers.

Most of the fucoidans extracted from sea cucumbers have →3)Fuc (1→ linked linear structure as shown in Figure 8. These fucoidans exhibit powerful lipid-modulatory effects. Fucoidan obtained from *I. badionotus* ameliorates serum levels of TC and TG in C57BL/6J mice fed with a high-fat high-sucrose diet at the dosage of 80 mg/kg/d (Wang et al., 2016). Of note, the fucoidan isolated from *P. graeffei* shows a better hypolipidemic effect than that from *I. badionotus* by increasing the expression of CYP7A1 in SD rats fed with a high-fat diet at the dosage of 40 mg/kg. The structure-activity relationship analysis suggests that 4-O-sulfation of the fucoidan obtained from *P. graeffei* benefits its lipid-lowering effect (Chen et al., 2012; Hu et al., 2015; Li et al., 2017). Furthermore, this fucoidan obtained from *P. graeffei* mainly act in the colon by increasing the abundance of *Bacteroidetes* and *Actinobacteria* and reducing the abundance of *Firmicutes* and *Proteobacteria* (Li et al., 2018b). Similar to FCS, fucoidan from the sea cucumber *A. molpadioides* inhibits adipocyte proliferation and differentiation as evaluated in 3T3-L1 cells and mice at the dose of 200 μg/ml and 80 mg/kg/d, respectively, via enhancing the expression of Wnt/β-Catenin signaling molecules, such as Wnt10b, β-Catenin, Fz, and LRP5, which suppress the expression of transcriptional factors including SREBP-1c, PPARγ, and C/EBPα, and their downstream genes such as FAS and glycerol-3-phosphate acyl-transferase (Yu et al., 2014a; Xu et al., 2014).

A recent study indicates that fucoidans extracted from *T. ananas* with low Mw and a random coil conformation have better effect than those with large Mw on attenuating hyperlipidemia and fat accumulation (Yu et al., 2014b; Xu et al., 2018; Zhu et al., 2021). Another study also demonstrates that the sulfated polysaccharides containing FCS and fucoidan that are isolated from sea cucumber *Stichopus japonicus* as well as its depolymerized derivatives decrease serum levels of TC and TG and fat accumulation in BALB/c mice fed with a high-fat diet by modulating gut microbiota at the dosage of 300 mg/kg/d. In addition to increasing the ratio of *Bacteroidetes*/*Firmicutes*, these polysaccharides enhance the abundance of the genus *Akkermansia* in the phylum Verrucomicrobia, which is associated with weight reduction. Furthermore, these polysaccharides increase the SCFAs-producing microbiota including *Bacteroides* and *Alloprevotella* as well as the content of SCFAs, which benefit glucose tolerance and insulin resistance. Of note, the depolymerized derivatives with similar structural characteristics as its native polysaccharides show better effect on preventing fat accumulation. These derivatives improve the enrichment of health-beneficial bacteria including *Akkermansia muciniphila* and *Parabacteroides goldsteinii*, suggesting that fucoidans with low Mw are superior to the growth of beneficial microbiota (Zhu et al., 2018a). These polysaccharides extracted from sea cucumber *S. japonicus* and its depolymerized derivatives also exhibit similar effects in mice fed with a normal chow diet (Zhu et al., 2018b). Presently, the hypolipidemic bioactivity of the →3)Fuc (1→ linked linear fucoidan isolated from *Ludwigothurea grisea* and *Holothuria tubulosa* as well as the →3,4)Fuc (1→ branched fucoidan extracted from sea cucumber *Apostichopus japonicus* has been reported (Figure 8) (Mulloy et al., 1994; Yu et al., 2015; Chang et al., 2016). The mechanisms of action of fucoidans are shown in Figure 6.

Glycosaminoglycans (GAGs) extracted from sea cucumber *Metriatyla scabra* also reduce serum levels of TC and LDL-c as well as atherosclerosis index and enhance HDL-c level via inhibiting HMGCR and improving the activity of LPL. These GAGs show dose-dependent effects when the dosages are lower than 20 mg/kg (Liu et al., 2002). The polysaccharides isolated from sea cucumber *Holothuria leucospilota* (HLP) are rich in sulfated GAGs. These polysaccharides with an oral dosage of 300 mg/kg/d reduce plasma levels of TC, TG, and LDL-c, and enhance the levels of HDL-c and SCFAs in male BALB/c mice. Mechanistically, HLP improves the expression of PPARα and CD36 as well as the abundance of gut microbiota. Accumulating studies have demonstrated that the reduced ratio of *Bacteroidetes*/*Firmicutes* promotes the development of obesity in different models (Zhu et al., 2018c). At the phylum level, HLP elevates the amount of *Bacteroidetes*, *TM7*, *Cyanobacteria* and *Tenericutes* and reduces the abundance of *Firmicutes*, *Proteobacteria*, *Spirochaetes*, and *Actinobacteria*. Of note, HLP intervention increases the abundance of SCFAs-producing bacterial genera including *Clostridium*, *Turicibacter*, *Allobaculum*, and *Ruminococcus* (Zhao et al., 2020). In line with these findings, gastrointestinal digestion reduces the Mw and changes the microstructure of polysaccharides that are extracted from *H. leucospilota in vitro* (Yuan et al., 2019a). Furthermore, these polysaccharides (200 mg/kg BW) are found to ameliorate hyperlipidemia in rats via reducing the expression of ACC and CD36 and potentially by enhancing the production of SCFAs (Yuan et al., 2019b). Although the structural characteristics of GAGs isolated from *A. japonicus* and *H. tubulosa* have been elucidated, their hypolipidemic effects have not been investigated (Yang et al., 2015; Ustyuzhanina et al., 2018; Chen et al., 2019). The mechanisms of action of carbohydrates derived from sea cucumbers are summarized in Figure 6.

### Protein and peptide

A recent literature demonstrates that the sea cucumber ovum powder, which mainly composed of protein (62.08%) as well as other components such as polyunsaturated FAs (6.74%) can significantly reduce plasma TG and hepatic TG and TC, thereby improving NAFLD in rats fed with a high-fat diet at the dosage of 450 mg/kg (Han et al., 2020). Mechanistically, this powder modulates the relative expression of 767 proteins as revealed by LC-MS/MS. Some of these differentially expressed proteins are associated with FA oxidation and lipogenesis (Han et al., 2020). However, the contribution of other lipid-lowering components contained in this sea cucumber powder cannot be ruled out. Therefore, the actual hypolipidemic effects of sea cucumber proteins need to be further investigated in future.

**FIGURE 9:**
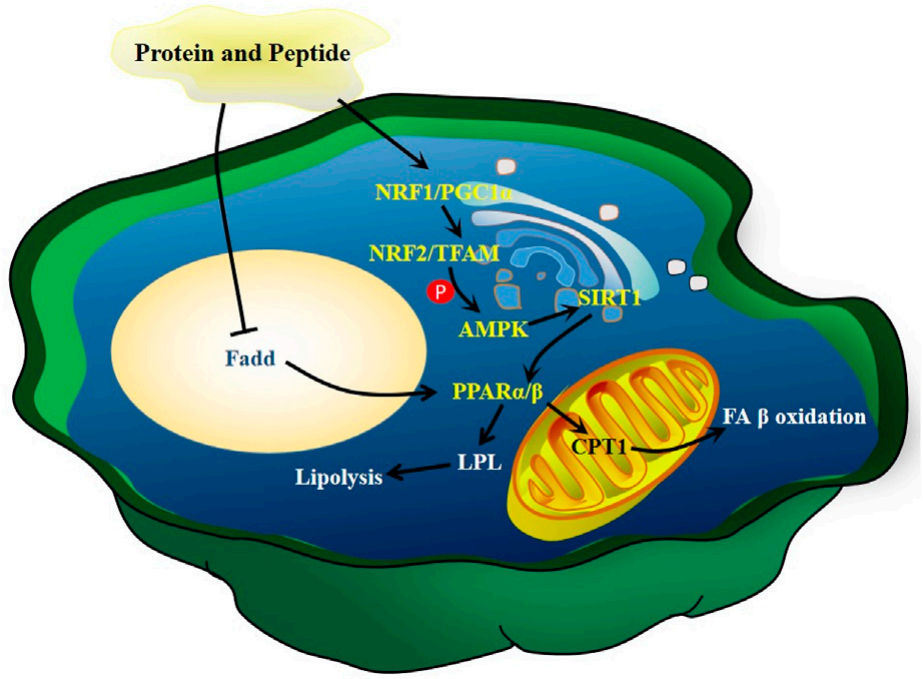
The mechanisms of action of peptides and proteins of sea cucumbers. Fadd, FAS associated death domain protein; NRF, nuclear respiratory factor; TFAM, mitochondrial transcription factor A.

Diet supplementation of collagen peptides (2.4 g/kg) isolated from *C. frondosa* reduces serum TG in rats (Hu X. Q. et al., 2012). The peptides obtained from sea cucumber *S. japonicus* enhance gluconeogenesis and maintain lipid homeostasis via increasing the mRNA expression of *AMPK*, *PPARγ coactivator 1-α* and its downstream genes *PPARα* and *PPARβ* in a dose-dependent manner in the liver, skeletal muscle, and heart. Furthermore, these peptides enhance the expression of *LPL* and *CPT1*, which are key rate-limiting enzyme genes for lipoprotein hydrolysis and FA β-oxidation, respectively (Yu et al., 2020). The peptides isolated from sea cucumber *Stichopus japonicas* can increase the content of unsaturated lipids in mouse and rat hippocampus (Lu et al., 2022a). As unsaturated lipids can modulate lipid metabolism (Djuricic and Calder, 2021; Mitrovic et al., 2022), the above results suggest that peptides alleviate hyperlipidemia by regulating the metabolism of unsaturated lipids. The potential mechanisms of action of peptides and proteins of sea cucumbers are summarized in Figure 9.

## The sea cucumber-devived coumpounds for treatment of cardiac complications

In addition to lipid-lowering, sea cucumber-derived compounds have other attractive bioactivities for treatment of cardiac complications. Studies on system pharmacology predict that sea cucumber-derived compounds have potential application for treatment of cardiac complications (Guo et al., 2015). Theses acitivites inculude anti-hypertensive, anti-angiogenic, anti-inflammatory, anti-diabetic, anti-coagulation, and antioxidation activities. As these activities have been reviewed by different groups, we make a brief review of these aspects in the following (Bordbar 2011; Khotimchenko, 2018; Hossain et al., 2020).

The cerebrosides and long-chain bases from *A. molpadioides* can reduce serum glucose level in addition to improving the lipid profiles in high fat diet-fed mice (Liu et al., 2015). A study demonstrates that sea cucumber-derived ceramides and glucosylceramides attenuate insulin resistance in high-fructose-diet-induced rats via upregulation of the insulin receptor substrate-phosphatidylinositol 3 kinase-serine/threonine kinase signalling pathway (Yang et al., 2021). As reviewed recently, the anti-diabetic effects of sea cucumber-derived peptides and carbohydrates are related to their modulation of angiogenesis (Khosravifar et al., 2022).

The sea cucumber-dereived carbohydrates and other components are able to inhibit clot and thrombus formation as reviewed previously (Pomin, 2014; Dashi and Naiwal, 2021). The mechanisms of action of these components have also been documented (Dashi and Naiwal, 2021). For instance, a clinical study has demonstrated that the Kang-Shuan Capsule, which consisted of *Holothuria ldeucospilota* acid mucopolysaccharide, improves lipid profiles, reduces blood viscosity, and exhibits good anticoagulant effect in patients with ischemic heart disease (Wang et al., 1997).

CVD is closely associated with nuclear factor kappa B (NF-κB)-mediate inflammatory response (Zhang Q. et al., 2022). Many sea cucumber-derived bioactive compounds including Ds-echinoside A, Frondoside A, Holothurin A1, and Psolusodie are found to inhibit NF-κB. As reviewed recently, sea cucumber compounds can target the NF-κB signalling pathway, which is involved in inflammation, immunity, cellular differentiation, cell adhesion, and survival (Wargasetia 2022). The extracts of *Holothuria forskali* and *Parastichopus tremulus* are reported to inhibit inflammation in endothelial cells (Mena-Bueno et al., 2016). In a triple-blinded randomized controlled trial, the *Stichopus horrens* extract toothpaste can alleviate plaque-induced gingivitis, suggesting the anti-inflammatory effect of the sea cucumber compounds in human (Bakar et al., 2020). The oligopeptides obtained from *A. japonicus* and *A. leucoprocata* may supress inflammatory response via inhibiting Toll-like receptor 4/myeloid differentiation factor 88/NF-κB signalling pathway (Wan et al., 2020). The fucoidan from *A. japonicus* reduces lipopolysaccharide-induced inflammation via inhibiting the phosphorylation of p38 mitogen-activated protein kinase/extracellular regulated protein kinase1/2 and the downstream NF-κB in mice (Yin et al., 2019). The anti-inflammatory effects of the carbohydrates obtained from sea cucumbers have been documented by various groups of researchers (Kapoor et al., 2019; Xu et al., 2022).

The anti-oxidant compounds, such as peptides, from sea cucumbers may also benefit CVD because the initiation and development of CVD are tightly associated with oxidative stress (Lu et al., 2022b; Qiao et al., 2022). The Reinhartdt and Sea Cucumber Capsule improves agitation in moderate to severe Alzheimer disease partly due to its effect on attenuating oxidative stress (Yu et al., 2017). The aqueous extract of sea cucumber *Holothuria atra* exhibits anti-oxidation activity *in vitro* and in doxorubicin-induced rats (Ibrahim et al., 2017). The saponins from *Holothuria lessoni* contribute to the anti-oxidant activity of this sea cucumber extracts (Bahrami et al., 2018). The health-beneficial effects including antioxidant, anti-diabetes, and immunomodulatory activities of sea cucumber peptides have been recently reviewed (Lu et al., 2022a).

Additionally, sea cucumber is an important constituent of food trerapy for treatment of hypertention in Traditional Chinese Medicine (Zou, 2016). The peptides obtained from *Acaudina molpadioidea* and *Actinopyga lecanora* show angiotensin I-converting enzyme inhibitor effect and exhibits antihypertensive activity in rats at 3 μM/kg and 800 mg/kg, respectively (Zhao et al., 2009; Vishkaei et al., 2016; Festa et al., 2020).

## Conclusion and future perspetive

The components of sea cucumbers exhibit powerful lipid-lowering activity and have great potential applications for intervention of CVD and NALFD via modulating multiple signaling pathways involved in lipid homeostasis as well as gut microbiota. Saponins are one of the most important hypolipidemic components of sea cucumbers. Saponins of sea cucumbers enhance the LXR, SREBP-1c, and PPARα signaling pathways. Among saponins isolated from sea cucumbers, only the lipid-lowering effects and mechanisms of action of echinoside A have been well-documented. Other bioactive compounds derived from these saponins need to be evaluated in the future to understand their potential structure-activity relationship. FAs and phospholipids are well-documented lipid-lowering components of sea cucumbers. EPA, PC, and PE exhibit powerful hypolipidemic effects, and the combination of EPA-PC or EPA-PE shows attractive applications for treatment of NALFD. Long-chain bases are the main active structural units of cerebrosides for intervention of hyperlipidemia. These long-chain bases modulate multiple signaling pathways involved in lipid homeostasis as well as gut microbiota. However, the studies of the lipid-lowering activity of these lipids are mostly carried out in NAFLD models. The activity of these compounds needs to be investigated in CVD models in the future. Carbohydrates derived from sea cucumbers also have a potential application for treatment of hyperlipidemia. Structure-activity relationship analysis demonstrates that 4-O-sulfation of the glycosyls as well as low Mw polysaccharides benefit their hypolipidemic activities. However, comparative studies need to be performed to investigate the relationship between Mw and the lipid-lowering effect of these carbohydrates.

## Glossary

ABC: ATP binding cassette
ACC: acetyl CoA carboxylase
ACOX1: acyl-CoA oxidase 1
AMPK: adenosine monophosphate activated protein kinase
Apo: apolipoprotein
ATGL: adipose triglyceride lipase
CD36: cluster of differentiation 36
C/EBPα: CCAAT/enhancer binding protein-α
CPT: carnitine palmitoyl transferase
CVD: cardiovascular disease
CYP7A1: cholesterol 7-α-hydroxylase A1
EPA: eicosapentaenoic acid
EPA-PC: EPA-enriched phosphatidylcholine
EPA-PE: EPA-enriched phosphatidylethanoamine
FA: fatty acid
FAS: fatty acid synthase
FCS: fucosylated chondroitin sulfate
Fz: frizzled receptor
GAGs: glycosaminoglycans
G6PDH: glucose-6-phosphate dehydrogenase
HDL-c: high-density lipoprotein cholesterol
HLP: polysaccharides isolated from sea cucumber Holothuria leucospilota
HMGCR: 3-hydroxy-3-methyl-glutaryl-CoA reductase
HSL, hormone-sensitive lipase;
LC-MS/MS: liquid chromatography-tandem mass spectrometry
LDL: low-density lipoprotein
LDL-c: low-density lipoprotein cholesterol
LPL: lipoprotein lipase
LRP: lipoprotein receptor-related protein
LXR: liver X receptor
Mw: molecular weight
NAFLD: non-alcoholic fatty liver disease;
PlsCho: plasmanyl phosphatidylcholine
PlsEtn: plasmenyl phosphatidylethanoamine
PPAR: peroxisome proliferator-activated receptor
SCD: stearoyl-CoA desaturase
SCFA: short-chain fatty acid
SD: Sprague-Dawley
SREBP: sterol regulatory element-binding protein
TC: total cholesterol
TG: triglyceride
